# XPO1/Exportin-1 in Acute Myelogenous Leukemia; Biology and Therapeutic Targeting

**DOI:** 10.3390/biom15020175

**Published:** 2025-01-24

**Authors:** Øystein Bruserud, Frode Selheim, Maria Hernandez-Valladares, Håkon Reikvam

**Affiliations:** 1 Acute Leukemia Research Group, Department of Clinical Science, University of Bergen, 5021 Bergen, Norway; mariahv@ugr.es (M.H.-V.); hakon.reikvam@uib.no (H.R.); 2 Section for Hematology, Department of Medicine, Haukeland University Hospital, 5007 Bergen, Norway; 3 Proteomics Unit of University of Bergen (PROBE), University of Bergen, Jonas Lies Vei 91, 5009 Bergen, Norway; frode.selheim@uib.no; 4 Department of Physical Chemistry, University of Granada, Avenida de la Fuente Nueva S/N, 18071 Granada, Spain; 5 Instituto de Investigación Biosanitaria Ibs. Granada, 18012 Granada, Spain

**Keywords:** Exportin 1, XPO1, acute myeloid leukemia, selinexor, RNA, therapeutic targeting, clinical studies

## Abstract

Exportin 1 is responsible for the export of hundreds of proteins, several RNA species and ribosomal components from the nucleus to the cytoplasm. Several transported proteins are important for regulation of cell proliferation and survival both in normal and malignant cells. We review the biological importance and the possibility of therapeutic targeting of Exportin 1 in acute myeloid leukemia (AML). Exportin 1 levels can be increased in human primary AML cells, and even exportin inhibition as monotherapy seems to have an antileukemic effect. The results from Phase I/II studies also suggest that exportin inhibition can be combined with conventional chemotherapy, including intensive induction and consolidation therapy possibly followed by allogeneic stem cell transplantation as well as AML-stabilizing therapy in elderly/unfit patients with hypomethylating agents. However, the risk of severe toxicity needs to be further evaluated; hematological toxicity is common together with constitutional side effects, electrolyte disturbances, and gastrointestinal toxicity. A recent randomized study of intensive chemotherapy with and without the Exportin inhibitor selinexor in elderly patients showed reduced survival in the selinexor arm; this was due to a high frequency of relapse and severe infections during neutropenia. Experimental studies suggest that Exportin 1 inhibition can be combined with other forms of targeted therapy. Thus, Exportin 1 inhibition should still be regarded as a promising strategy for AML treatment, but future studies should focus on the risk of toxicity when combined with conventional chemotherapy, especially in elderly/unfit patients, combinations with targeted therapies, identification of patient subsets (AML is a heterogeneous disease) with high susceptibility, and the possible use of less toxic next-generation Exportin 1 inhibitors.

## 1. Introduction

Acute myeloid leukemia is an aggressive and heterogeneous hematological malignancy characterized by bone marrow infiltration of immature malignant myeloid cells; many patients additionally have high levels of circulating leukemic cells, but few patients have extramedullary organ manifestations [1,2,3]. The leukemic cells are characterized by increased proliferative capacity, high antiapoptotic signaling, and also limited signs of lineage differentiation [1,2] even though limited morphological and molecular signs of neutrophil, monocytic, erythroid or megakaryocytic differentiation can be present in some patients, whereas others have a stem cell-like AML cell phenotype [2].

Many patients survive only for a few months after diagnosis if they do not receive effective AML-directed therapy [3,4,5]. Younger patients fit for intensive treatment with high-dose cytotoxic drugs possibly combined with allogeneic stem cell transplantation can be cured [3]. Less intensive and thereby less toxic therapies (e.g., hypomethylating agents plus venetoclax) may also induce complete remissions and thereby represent an alternative bridge to (reduced intensity) allotransplantation [3,5]. However, many unfit and elderly patients still receive only AML-stabilizing treatment and have a median survival of 10–15 months [3,4,5,6]. Thus, there is a need for more effective and less toxic therapeutic strategies to increase the number of cured patients after intensive therapy and to improve survival for patients who can only receive AML-stabilizing treatment.

Exportin 1 (also referred to as XPO1 or CRM1) is a nuclear receptor that is involved in the export of a wide range of proteins, including many tumor suppressors and oncoproteins as well as RNA species [7,8]. It is overexpressed in various malignant cells and is responsible for the nuclear export of many molecules that are involved in carcinogenesis/leukemogenesis. A recent study has even described a prognostic impact of Exportin 1 expression in AML [9]. For this reason, Exportin 1 is regarded as a possible therapeutic target in cancer therapy. This is true also for AML, but it should be emphasized that Exportin 1 inhibition is not a part of routine AML therapy and therefore should be tried only for patients included in clinical studies.

## 2. Methodology for Preparing the Review

The review is based on articles included in the PubMed database. The articles were selected after literature search using the key words/key word combinations Exportin 1, Exportin 1 inhibition, selinexor, acute myeloid leukemia, acute myeloid leukemia + Exportin 1, and acute myeloid leukemia + selinexor.

## 3. The General Function of Exportin 1; Nuclear Export of Various Proteins and RNA

The general function of Exportin 1 is molecular transport from the nucleus to the cytoplasm. It should be emphasized that the general functions of Exportin 1 have been characterized in detail mainly in other cell types than AML cells.

### 3.1. Protein Export

**Figure 1:**
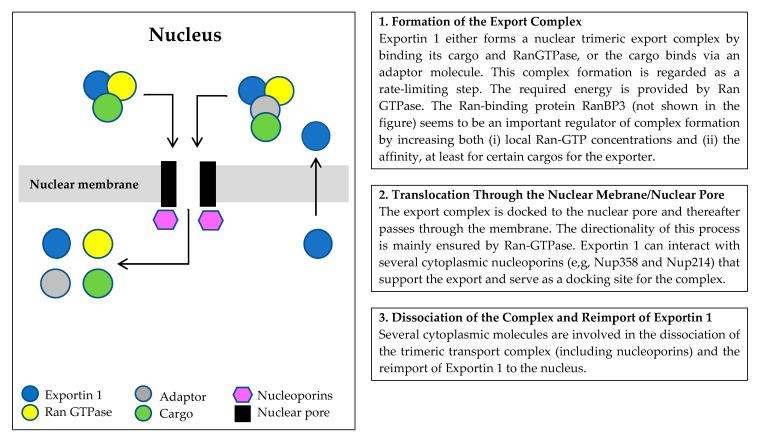
Nuclear molecular export through the nuclear membrane/nuclear pore by Exportin 1; a simplified overview of the three steps and important molecular interactions. Exportin 1 can carry a large number of cargo molecules, including both proteins (e.g., oncoproteins, tumor suppressors), ribonucleoproteins and various RNA species.

Exportin 1 is a nuclear receptor involved in the export of a wide range of proteins [7,8] through a three-step process (Figure 1) [10]:

- Formation of the export complex. Exportin 1 forms a nuclear trimeric export complex by binding its export cargo and RanGTPase. This complex formation can be regulated by the phosphorylation status of the cargo [11,12] and is regarded as a rate-limiting step [13]; the required energy is provided by Ran GTPase. The Ran-binding protein RanBP3 seems to be an important regulator of complex formation by increasing (i) local Ran-GTP concentrations [14] and (ii) the affinity at least for certain cargos for the exporter [15,16].
- Translocation through the nuclear pore. The export complex is docked to the nuclear pore and thereafter passes through the nuclear membrane. The directionality of this process is mainly ensured by Ran-GTPase [10]. Exportin 1 can interact with several cytoplasmic nucleoporins (including Nup358 and Nup214) that support the export and serve as a docking site for the complex [10,17].
- Dissociation of the complex. Several cytoplasmic molecules are involved in the dissociation of the trimeric transport complex (including nucleoporins) and the reimport of Exportin 1 to the nucleus [10].

Exportin 1 is thereby important for the nuclear export of a wide range of client proteins, including nearly 220 proteins with a structural nuclear export signal (NES) [18]. However, a recent study suggests that the number of client proteins in humans is more than 1050 [19]. Many of these client proteins probably bind to Exportin 1 via various adapter molecules, and they include tumor suppressors (e.g., p53, p27), many oncogene proteins (e.g., cyclins) and important regulators of fundamental cellular processes, including intracellular vesicle formation, cytoskeleton functions, ribosome maturation and translation, and mRNA degradation [7,18,19].

### 3.2. Nuclear Export of Small RNAs

Exportin 1 is important in the nuclear export of multiple RNA species. Its role in the export of ribonucleoprotein complexes is described in Section 4.2, whereas its importance for the export of various small RNA species [7,20,21] is described below.

Messenger RNA (mRNA). mRNA is transported either by the main NXF1-mediated pathway or the more selective Exportin 1 pathway [20]. This Exportin 1-dependent export is possible by RNA binding to various adaptor proteins and includes export of mRNAs for several oncoproteins [20,21].

The eukaryotic translation initiation factor 4E (eIF4E) binds selected mRNAs and thereby controls gene expression through effects on the nuclear export, including several genes important for the regulation of cell cycle progression and cellular survival [22,23]. Several of these mRNAs also seem to be important for the progression of malignant diseases [20]. eIF4E then associates with mRNAs containing a specific binding motif to form specific ribonucleoproteins before Exportin 1 dependent export [20,21,24,25,26]. However, other adaptor proteins can also bind mRNAs to Exportin 1 and thereby initiate nuclear export, including Leucine-rich pentatricooeotide repeat protein (LRPPRC), RNA binding protein human antigen R (HUR), and nuclear export factor 3 (NXF3) [25,26].

Small nuclear RNAs (snRNA). These small RNAs have a role in mRNA splicing, and Exportin 1 thereby becomes involved in mRNA splicing through its regulation of snRNA maturation [7,20]. The snRNAs form complexes with various adaptor proteins and Exportin 1; after export to the cytoplasm the snRNAs are released and modified before they form new import complexes that are shuttled back to the nucleus and further modification within the nucleolar Cajal bodies (i.e., membrane-less protein/RNA-containing organelles) before they become parts of the spliceosome (see [20,24]).

Micro RNA (miRNA) and transfer RNA (tRNA). miRNAs are important for posttranscriptional regulation of gene expression, whereas tRNAs are involved in transport of amino acids to the ribosomal complex through their function as adaptors between mRNA and the synthesized protein chain [25]. The miRNA and tRNA precursors are mainly exported by Exportin 5 and Exportin t, respectively [21]. However, Exportin 1 represents an alternative export mechanism for both these RNA forms [27,28,29,30], and this alternative export seems to be an alternative pathway for genesis of certain mature miRNAs [27,28,29].

To summarize, Exportin 1 inhibition will probably influence the biogenesis and biological function of diverse small RNA species.

## 4. The General Function of Exportin 1; Regulation of Mitosis and Ribosome Biogenesis

### 4.1. Control of Mitosis

The centromere links the two chromatids together during mitosis, and the kinetochore is then a complex multiprotein structure that assembles on the centromere and links chromosomes to the mitotic spindle [31,32,33,34,35]. Thus, the kinetochore is thereby involved in chromosomal segregation during mitosis together with several other proteins/complexes including RanBP2, RanGAP1, RanGTP [36], the centrosomal scaffold protein pericentrin [37], the γ-tubulin ring complex [38,39], and nucleophosmin [40,41].

Exportin 1 targets a molecular complex including RanBP2, RanGAP1 and RanGTP to the kinetochore and thereby seems to stabilize the kinetochore-microtubules connection [32,33,42,43]. Furthermore, Exportin 1 is present at the centrosome throughout the cell cycle and seems to be involved in the recruitment of pericentrin; this scaffold protein recruits the γ-tubulin ring complex, and these proteins/complexes then function to nucleate microtubules during the early steps of mitotic spindle formation [32,33,44].

Thus, Exportin 1 seems important in the regulation of mitosis through its targeting of key proteins to specific steps in the mitotic spindle formation [32,33].

### 4.2. Ribosome Biogenesis

Exportin 1 facilitates the nuclear export of both the small (40S) and large (60S) ribosome subunits [45]. The formation of these subunits involves the synthesis of structural ribosomal RNA (rRNA) together with ribosomal proteins; these components then form nucleolar preribosomal subunits that are exported by Exportin 1 before they undergo further processing and gain translational capacity [46]. The preribosomal subunits then bind to Exportin 1 via the NMD3 adaptor protein [23,46]. Exportin 1 inhibition will therefore inhibit 28S rRNA processing and pre-47S rRNA synthesis [8,47]. Finally, cell line studies have demonstrated that Exportin 1 inhibition downregulates a wide range of ribosomal proteins [8,18,48,49].

## 5. Prognostic Impact of Exportin 1/XPO1 Levels in Human AML; Resistance to Conventional Chemotherapy, *XPO1* Mutations, and Cellular Exportin 1 Compartmentalization

### 5.1. The Prognostic Impact of Exportin 1 Expression in AML Patients Receiving Conventional Intensive Treatment

Exportin 1 is upregulated in a wide range of solid tumors and is associated with an adverse prognosis in many of these malignancies (for references see [45]). The molecular mechanisms behind cancer-associated Exportin 1 upregulation have not been characterized in detail but seem to involve c-Myc as a positive regulator and p53 as a negative regulator of its expression [50]; both these molecules are also client proteins of Exportin 1 [44]. c-Myc seems to cause a transcriptional Exportin 1 upregulation as a part of a broader transcriptional program also upregulating several ribosomal proteins [23,51]; the Exportin 1 upregulation may then represent a coordination of c-Myc-induced increased transcription with increased capacity of nuclear export of c-Myc-increased RNAs.

The AML cell expression of Exportin 1 has been compared with the expression of normal CD34^+^ bone marrow cells [9]. The protein levels for 511 newly diagnosed AML cell samples were then compared with 21 normal CD34^+^ cells. When comparing the overall results, the authors observed that there was no statistically significant difference between the leukemic and normal cells, but the AML cells showed a wider variation with 21% of the patients showing higher and 12% showing lower levels than the normal CD34^+^ bone marrow cells. Furthermore, the Exportin 1 levels were higher in patients with *FLT3* mutations, and the Exportin 1 levels showed significant correlations with proteins involved in AKT signaling, including (i) AKT itself; (ii) the upstream mediators phosphatidylinositol-3 kinase p85 (PI3Kp85), phospho-phosphatase, and tensin homolog (phospho-PTEN); and (iii) the downstream phospho-BCL2-associated agonist of cell death (phospho-BAD) (Ser112, Ser136) and 14-3-3. The association with p53/MDM2 seems to be more complex, with no significant correlations with p53 or MDM2, but p53 levels were highest for patients with high Exportin 1 and low MDM2 levels.

Other studies have shown that Exportin 1 levels are also relatively high in *PICALM-MLLT10*-fusion-positive [52] and probably also in *DNMT3A*-mutated AML [53].

These authors also investigated the possible prognostic impact of Exportin 1 levels for their 511 patients with newly diagnosed AML, but the antileukemic treatment was unfortunately not described in detail [9]. Exportin 1 protein expression showed a more than 30-fold variation between patients, and high Exportin 1 levels were especially observed in patients with high p53 and low MDM2 (mouse double minute 2) levels. High Exportin 1 expression had an adverse prognostic impact and was associated with short survival after intensive antileukemic therapy, and this adverse impact remained in multivariate analysis including age, albumin level, white blood cell count, karyotype and Exportin 1 level. These authors also investigated the in vitro sensitivity to Exportin inhibition for a subset of 46 patients (age 21 to 85 years, 16 patients above 70 years of age); *TP53* mutation was then associated with a weak proapoptotic effect of Exportin 1 inhibition, whereas *FLT3* mutations were associated with high sensitivity. Thus, Exportin 1 expression seems important for clinical chemosensitivity in human AML, and studies in AML cell lines as well as primary AML cell samples suggest that there is a crosstalk between p53 and Exportin 1, especially with regard to regulation of apoptosis; this crosstalk seems less important with regard to the antiproliferative effect of Exportin 1 inhibition.

### 5.2. Overcoming Resistance to Conventional Cytotoxic Drugs

Experimental studies suggest that there is a synergistic effect between Exportin 1 inhibitors and anthracyclines in head and neck squamous cell carcinomas, i.e., Exportin 1 inhibition can reverse anthracycline resistance [54]. The same seems to be true for topoisomerase II inhibitors in myeloma cells [55]. XPO1 inhibitors may also overcome the resistance for several other anticancer drugs [8]. Both anthracyclines and topoisomerase II inhibitors are used in the treatment of AML [3,5], but it is not known whether Exportin 1 inhibitors can be used to overcome conventional drug resistance in human AML cells.

### 5.3. Exportin 1/XPO1 Point Mutation in E571K Substitution and Altered Compartmentalization

A recurrent *XPO1* point mutation (NM_003400, chr2:g61718472C>T) resulting in an E571K substitution has been described in various B cell malignancies and in Hodgkin’s lymphoma [56,57,58]. This substitution is harbored within the hydrophobic groove of the Exportin 1 protein, but the effect of the mutation on its export capacity seems to be limited [59], and the sensitivity to Exportin 1 inhibitors does not seem to be altered either [57]. However, the mutation seems to have an effect on the subcellular localization of Exportin 1, with a higher localization in the cytoplasm for cells with the E571K mutation compared to *XPO1*-wt and *XPO1* with the E571G mutation that has also been detected in malignant B cells [56]. Finally, the mutant XPO1 seems to modulate the nuclear export/import balance of relevant cargoes through binding to importin β1 [56].

To the best of our knowledge, it is not known whether these XPO1 mutations and XPO1 mislocation can be detected or have any clinical impact in human AML. However, a recent study described the generation of an induced pluripotent stem cell line from an AML patient; this cell line had maintained mutations of XPO1 as well as PALB2, and it showed characteristics similar to embryonic stem cells [60]. This observation suggests that XPO1 mutations have the capacity to contribute to malignant transformation also in AML, but additional studies are definitely needed.

## 6. Exportin 1 Mediated Export of Small Noncoding (snc) RNAs in Human AML; Long Non-Coding RNAs Seem Especially Important for the Antileukemic Effect of XPO1 Inhibitors, but Effects on Other sncRNAs May Also Contribute

Exportin 1 is important for the export of various noncoding small RNAs from the nucleus (see Section 3.2). This seems to be a general function of Exportin 1, and it is probably important also in AML cells several studies suggest that non-coding RNAs are important both for leukemogenesis and chemosensitivity in human AML cells:

- The prognostic impact of long non-coding RNAs (lncRNA) in AML cells was investigated for young adults (aged <60 years) with de novo normal karyotype AML [61]. The authors constructed a prognostic score based on the analysis of a training patient population that identified 24 lncRNAs associated with event-free survival. High scores had an independent prognostic impact were associated with shorter disease-free and event-free survival. Furthermore, double *CEBPA* mutations, *NPM1* mutations and *FLT3-ITD* were associated with distinct lncRNA profiles.
- Expression of the lncRNA KIAA0125 was compared for AML bone marrow cells from 347 de novo patients [62]. Higher KIAA0125 expression was associated with RUNX1 mutation but inversely correlated with the t(8;21) karyotypes. Furthermore, high KIAA0125 expression was associated with a reduced complete remission rate as well as shorter overall and disease-free survival among 227 patients receiving intensive therapy; this prognostic impact was also observed in validation analyses and multivariable analysis. Finally, higher KIAA0125 expression was associated with an AML stem cell phenotype that had an adverse prognostic impact.
- The lncRNA expression was evaluated in bone marrow AML cells derived 148 untreated patients above 60 years of age with normal karyotype [63]. Distinctive lncRNA profiles were associated with *FLT3-ITD* and mutations in *NPM1, CEBPA, IDH2, ASXL1* and *RUNX1* genes. These authors also constructed a lncRNA score based on the lncRNAs most strongly associated with event-free survival in the 148 elderly patients. Patients with unfavorable lncRNA score had lower complete response rate as well as shorter AML-free and overall survival, and this adverse impact was confirmed in multivariate analyses and in a validation cohort.
- Levels of lncRNA and antisense non-coding RNA of the *INK4* locus (ANRIL) in bone marrow mononuclear cells were compared for 178 de novo AML patients and 30 healthy donors [64]. lncRNA ANRIL levels were increased in AML; high levels were especially associated with adverse *Flt3-ITD* and decreased levels with favorable inv(16). Furthermore, high lncRNA ANRIL was significantly associated with a lower remission rate and shorter event-free and overall survival even in multivariate Cox regression analyses.
- miR-34c expression in primary AML cells derived from 122 patients with de novo AML compared with 62 normal hematopoietic cells; its expression was significantly generally downregulated in AML cells (*p* < 0.001) and particularly low level was associated with shorter overall survival even in multivariate analysis [65].
- Hypoxia upregulates miRNA-146a and the CXCR4 chemokine receptor is thereby downregulated in normal monocytes; hypoxia upregulates miRNA-146a also in monocytic AML but this is not followed by downregulation of CXCR4 expression [66]. This maintenance of high CXCR4 expression is associated with increased resistance to cytarabine CXCR4 ligation.
- RUNX1 mutations were analyzed in younger (<60 years of age; *n* = 175) and older (≥60 years of age; *n* = 225) patients with primary normal karyotype AML receiving intensive antileukemic therapy [67]. RUNX1-mutated patients had lower complete remission rates as well as shorter disease-free, overall and event-free survival, and the mutation was also associated with downregulation of miR-223 that is a promoter of myelopoiesis.
- Expression of miR-21 and its target PDCD4 (Programmed Cell Death 4) was compared for AML cells and normal hematopoietic cells [68]. AML cells often showed increased miR-21 protein levels together with decreased PDCD4 levels, especially in NPM1mutant AMLs.
- A study of single-agent low-dose decitabine included 53 patients above 60 years of age (median age 74 years) with untreated AML; 19 had secondary AML and 16 had complex karyotypes [69]; 19patients reached complete remission; 9 additional patients had no morphologic evidence of AML but incomplete blood count recovery (i.e., overall response rate of 64%). High pretreatment levels of miR-29b (known to target DNA methyltransferases) were associated with clinical response.
- The circular RNA Hsa_circ_0009910 (circ_0009910) is upregulated in AML bone marrow cells as well as in AML cell-derived exosomes involved in cell–cell communication [70]. It is a regulator of cellular proliferation, apoptosis, and cell cycle progression, and these effects seem to involve miR-5195-3p together with Bcl-2/Bax.

Based on the studies reviewed above [63,64,65,66,67,68,69,70] we conclude that various sncRNA species show altered levels in human AML cells compared with normal cells (lncRNA, miR-324c, miR-21, Hsa_circ_0009910). Altered cellular levels of certain sncRNAs (lncRNA, miR-223, miR-21) can also be associated with AML-associated cell genetic abnormalities (t(8;21), *FLT3-ITD*, mutations of *NPM1*, *CEBPA*, *IDH2*, *ASXL1*, *RUNX1*), and the responsiveness to noncoding RNAs can be altered in AML cells compared with normal cells (miRNA-146a). The levels of certain sncRNAs are also associated with prognosis both for patients receiving intensive and potentially curative treatment (lncRNAs, miR-34c) and less intensive AML-stabilizing therapy (miR-29b). Many of the sncRNAs described above are involved in the regulation of cellular proliferation, apoptosis and cell cycle progression (for details and references see [63,64,65,66,67,68,69,70]). It therefore seems likely that inhibition of nuclear export of sncRNAs contributes to the antileukemic effects of Exportin 1 inhibitors.

## 7. Increased XPO1 Expression/Activity in AML Cells Is Not a Part of the NUP214 Role in Leukemogenesis

Nup358/RanBP2, Nup214/CAN, and Nup88 are all components of the cytoplasmic face of the nuclear pore complex (see also Section 3.1); Nup88 localizes between the two others, whereas both Nup88 and Nup214 seem to mediate their attachment to the nuclear pore complex [17]. The localization of Exportin 1 at the cytoplasmic face of the nuclear envelope (i.e., its transport capacity) is Nup358 dependent. These three NUP molecules can be involved in leukemogenesis [71,72,73,74,75]:

- NUP214 is involved in leukemogenesis as part of the SET-NUP214 and DEK-NUP214 fusion proteins that disrupt nuclear export by inhibiting Exportin 1 [71]. Both these fusions can be detected in human AML although they are uncommon [71,72,73,74]; very few patients have been included in these clinical studies [72,73,74] and the observations therefore have to be interpreted with great care. Studies of the SET-NUP214 protein in transgenic mice suggest that this fusion protein is not sufficient alone for leukemic transformation, but it inhibits myeloid differentiation of hematological progenitors through altered promotor interactions, leading to modulated epigenetic regulation, especially of HOXA genes [75,76]. Thus, its contribution to leukemogenesis seems to be caused by epigenetic effects on chromatin and thereby transcription regulation rather than by modulation of XPO1 activity [72].
- *NUP98-NSD1* gene fusion is associated with a characteristic gene expression profile and an adverse prognosis in pediatric AML, but again, it must be emphasized that very few patients have been studied [74].

Even though studies of the AML erythroid OCIM2 cell line suggest that downregulation of NUP214 protects AML cells from apoptosis through altered nucleocytoplasmic balance of NF-κB [77], it seems justified to suggest the hypothesis that the role of NUP-containing fusion proteins in leukemogenesis seems to mainly depend on the fusion partner (e.g., altered transcriptional regulation) rather than modulation of Exportin 1 activity.

## 8. Molecular Interactions of Exportin 1 in Human AML Cells; Studies of Exportin 1-Associated Molecular Functions and Cellular Effects of Exportin 1 Inhibition

The biological importance of Exportin 1 in AML has been investigated in many experimental studies, and the key observations from several important studies are summarized in Table 1. Taken together these observations support the following concluding comments [9,53,78,79,80,81,82,83,84,85,86,87,88,89,90,91,92,93,94,95]:

- AML is a very heterogeneous disease; the available data suggest that Exportin 1 is important for leukemogenesis/chemosensitivity, and Exportin 1 inhibition is therefore regarded as a possible therapeutic strategy for several different AML subsets/genotypes. However, there may be exceptional variants that are less susceptible, one example being *p53*-mutated AML.
- Exportin 1 protein levels are associated with prognosis/chemoresistance; future studies should investigate whether the susceptibility to Exportin 1 inhibition is also associated with the AML cells’ level of Exportin 1.
- Exportin 1 inhibition seems to alter the regulation of several fundamental cellular functions, including the regulation of proliferation/cell cycle progression, survival/apoptosis, metabolic functions (e.g., glutathione metabolism and glycolysis), DNA repair and transcription/epigenetic modulation as well as the activation of intracellular signaling pathways. Several of these effects have been investigated only in certain AML subsets, and the wide variation of Exportin 1 inhibitor effects suggests that the main effect of this therapeutic approach differs between subsets of AML.
- The overall results summarized in Table 1 suggest that the effects of Exportin 1 inhibition in AML cells are extensive and complex; this is not unexpected when taking into account the large number of client proteins for Exportin 1. However, another possibility that may contribute to the large number of observed effects is that the effects vary between AML patient subsets and depend on the genetic abnormalities and/or the AML models used in the various studies. The observation of increased DNA damage in certain studies [89,90] but not in another study [79] is consistent with this last possibility.

Taken together, these observations suggest that the molecular mechanisms responsible for the contribution of Exportin 1 in leukemogenesis and the effect of Exportin 1 inhibition will vary between patients due to the biological heterogeneity of the AML cell biology between patients. If this is true, the optimal use of Exportin 1 inhibitors may also vary between patients—for example, with regard to optimal timing or the optimal combination of Exportin 1 inhibitors with various forms of conventional cytotoxic and/or new targeted therapies.

## 9. Selective Inhibitors of Nuclear Export

### 9.1. Effects of Selective Inhibitors of Nuclear Export on AML Cells

Selective inhibitors of nuclear export represent a new class of small-molecule pharmacological agents that are orally bioavailable and act through covalent/reversible modification of the cysteine-528 in the cargo-binding pocket of Exportin 1/XPO1 [84]. The agents thereby inhibit binding and nuclear export of cargo proteins [84], and the nuclear retention of these proteins seems to finally restore DNA damage surveillance and induce cell cycle arrest, differentiation and apoptosis [82,84,94]. Experimental studies suggest that these effects also include the AML-initiating cell subset, whereas the effects on normal hematopoietic cells seem to be weaker [82]. Furthermore, among the AML-relevant proteins showing such nuclear retention are p53, p21, p27, Foxo3, Rib, surviving, and NPM1, and there is in addition a degradation of certain proteins, including XPO1, c-KIT, and FLT3 [84]. The XPO1 inhibitor selinexor also upregulates the purinergic receptor P2Ry2 in AML cells and thereby activates PI3K-AKT signaling; inhibition of this pathway potentiates the anti-leukemic effects of selinexor in experimental models [80]. Finally, the XPO1 inhibitor effect on NPM1 seems important both for the antiproliferative effect, the G1 arrest and the induction of differentiation that has been detected both by morphological examination showing signs of monocytic or granulocytic AML cell differentiation and increased expression of the molecular markers CD11b and CD14 [94]. Thus, selinexor will also inhibit the export of several molecules (e.g., differentiation markers) that may influence leukemogenesis [94].

### 9.2. Selinexor and Other XPO1 Inhibitors

Four selective XPO1 inhibitors have been investigated in experimental models across a range of malignancies, i.e., KPT-185, KPT-251, KPT-330 (selinexor), and SL-801 (felesonexor). Selinexor has also been investigated in clinical AML studies (see below [96]). The second-generation inhibitor KPT-8602 has been designed to have increased reversibility and limited blood–brain barrier penetration (see [96] for details). KPT-8602 has only been investigated in preclinical AML studies; based on patient-derived xenograft models, it was concluded that this agent was active against both AML blasts as well as leukemia-initiating cells and had minimal toxicity to xenografted human CD34^+^ cells.

### 9.3. Selinexor Pharmacokinetics

The pharmacokinetics of selinexor were recently reviewed [97]. The pharmacokinetic observations are mainly based on an oral intake of 100 mg once weekly, 60 mg twice weekly, or 80 mg twice weekly:

- The drug is usually administered as tablets, but there is no significant difference between tablet and suspension [97,98]. The maximal concentration is reached 2–4 h after intake. The maximal concentration seems consistent over a wide range of doses, an observation suggesting dose-independent absorption [97]. The variation between patients with regard to pharmacokinetics seems to be smaller when using flat dosing compared with dosing based on body surface area [97].
- There is only a minor difference in absorption and distribution between fed and fasted patients [98].
- Selinexor shows high plasma binding [97] and extensive tissue penetration [97,99].
- The half-life of selinexor is 6–8 h [97]. The drug shows limited metabolism, and the original non-metabolized agent is the major circulating form [97]. The most common metabolite has approximately 10% of the original binding activity and reaches a circulation level corresponding to approximately 5% of the selinexor level. Selinexor seems to be excreted mainly via the hepatobiliary route.
- Renal and hepatic impairment does not seem to have any major impact on the pharmacokinetics of the selinexor [97]. There is a small difference between patients depending on sex and bode weight [99], but this difference has been regarded to have no clinical relevance [97].
- The exposure appears to be higher in pediatric patients compared with adults receiving comparable body-size-based doses [100], but only minor age-dependent differences have been observed for adults [97].
- No pharmacokinetic drug–drug interactions have so far been observed [99].

To summarize the most important selinexor observations, the pharmacokinetic studies suggest that flat dosing should be possible also in AML, and no pharmacokinetic observations suggest a risk of harmful drug interactions when combined with conventional anticancer treatment and using twice-weekly dosing.

### 9.4. Hematological and Nonhematological Toxicity of Selinexor Monotherapy in Cancer Patients

**Figure 2:**
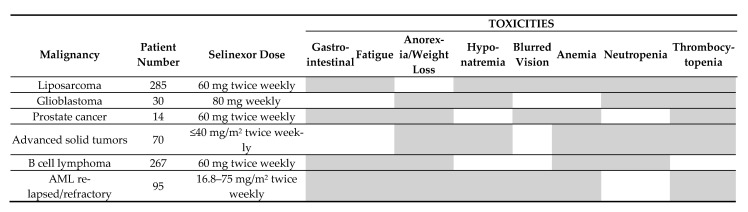
Nonhematological and hematological toxicity of selinexor monotherapy in various cancer patients; the figure indicates toxicities occurring in at least 20% of patients (indicated by grey color) with various solid tumors (liposarcoma [103], glioblastoma [104], prostate cancer [105]), advanced solid tumors [101], and hematological malignancies (B cell lymphoma [102], relapsed/refractory AML [106]). The left part of the figure states the malignant disease, number of patients included and selinexor dose for each of the six studies. The various toxicities are presented in the right part.

The toxicity of selinexor monotherapy for patients with various malignancies are summarized in Figure 2 [101,102,103,104,105,106,107]; the most important side effects being:

- Gastrointestinal. Gastrointestinal toxicity is very common and occurs in a majority of patients. This can be nausea/vomiting, decreased appetite, constipation, diarrhea, abdominal pain, or dysgeusia [98,103].
- Constitutional. Fatigue is most common, but weight loss is also frequent [102,103,104].
- Electrolytes and renal function. Electrolyte disturbances are common, especially hyponatremia.
- Eyes. Ocular side effects can occur, and blurred vision is most common [98,103]. According to a recent review [107] dry eyes are also relatively common. Rare cases of cataract progression have also been described, as has Meibomian gland dysfunction [107]. Due to the (small) risk of ocular toxicity, patients with certain ocular disorders have been excluded from some previous clinical studies of selinexor [108].
- Hematological. Hematological toxicity, including both neutropenia and thrombocytopenia, has been described in several studies.

Several less common toxicities have also been described, including circulatory, cardiac, respiratory, renal, and hepatic side effects. It should also be emphasized that several of these adverse effects of selinexor monotherapy (especially hematological toxicity) are similar to the adverse effects during conventional intensive AML chemotherapy. Selinexor is associated with similar toxicity when combined with conventional intensive AML therapy (see below, Section 9.5 and Table 2).

### 9.5. Nonhematological Toxicity for AML Patients Receiving Selinexor in Combination with Conventional Chemotherapy

Non-hematological toxicities are common in patients receiving selinexor in combination with conventional AML chemotherapy [108,109,110,111,112,113,114,115,116,117]; this is illustrated by the summarizing Table 2, which lists adverse events occurring in at least 10% of patients included in these studies. Many of these adverse events are common for patients receiving selinexor monotherapy and patients receiving conventional AML chemotherapy alone [108,109,110,111,112,113,114,115,116,117].

Only one randomized study of selinexor added to standard intensive AML therapy has been published [109]. This study compared frequencies of adverse events for intensive treatment with and without selinexor. Patients receiving selinexor showed increased frequency of ≥grade 3 nervous system toxicities (12% versus 2%) during induction cycles, and during the first consolidation there were increased frequencies of grade 3–4 cardiac, gastrointestinal (43% versus 26%), infectious (57% versus 37%), and metabolic/nutritional events (46% versus 29%). It was also a significant delay in neutrophil reconstitution (see below Section 10.5).

### 9.6. Hematological Toxicity of Selinexor in AML

The hematological toxicity in studies of selinexor combined with intensive AML chemotherapy is presented in Table 2 [109,110,111,112,113,114,115]; this is of particular importance because a large randomized study including elderly patients with newly diagnosed AML and receiving intensive chemotherapy showed decreased survival for patients receiving additional selinexor compared to patients not receiving selinexor [109]. The decreased survival was due to AML relapse/resistance as well as severe infections, and there was an increased time to neutrophil reconstitution after the induction cycles. A relatively long time until neutrophil reconstitution was also observed in two other clinical studies [113,114]. In our opinion, the question of hematological toxicity and especially the risk of severe neutropenia/infections must be carefully addressed in future clinical studies.

## 10. Clinical Studies of Selinexor Inhibition in AML; Review of the Antileukemic Efficiency of Monotherapy and Combined Treatment

### 10.1. Selinexor Monotherapy Can Have a Clinically Relevant Antileukemic Effect with Acceptable Toxicity Even in Relapsed/Refractory AML

A Phase I single-agent dose-finding study included 95 patients with relapsed/refractory disease (median age 70 years, range age 24–89 years of age) [106]. The study reported no dose-limiting toxicities or evidence for cumulative toxicity, and the recommended Phase 2 dose was selinexor 60 mg (corresponding to 35 mg/m^2^) twice weekly for two weeks in four-week cycles. The only non-hematological grade 3/4 toxicity occurring in >5% of patients was fatigue (14%). Other common toxicities were nausea (55%), diarrhea (40%), vomiting (38%), anorexia (55%), and less severe fatigue (44%). Furthermore, response evaluation was available for 81 patients; five patients reached complete hematological remission, two patients reached complete response with incomplete recovery of peripheral blood counts and one patient morphological AML-free state. Eight patients had stable disease for at least three months. Finally, an objective response was associated with increased progression-free (5.1 versus 1.3 months) and overall survival (9.7 versus 2.7 months) compared with nonresponders. The authors concluded that the drug had a clinically relevant antileukemic effect with acceptable toxicity.

A single-center, single-arm, Phase 2 study included 25 adults with high-risk myelodysplastic syndromes (MDS, 21 patients) or AML with 20–30% bone marrow blasts (4 patients) refractory to hypomethylating agents [116]. Each cycle consisted of selinexor 60 mg twice weekly for two weeks followed by one week without treatment. Six patients responded to the therapy with AML-free bone marrow as determined by morphological examination, and 12 additional patients achieved stable disease. The most common Grade 3/4 adverse events were thrombocytopenia in eight patients and hyponatremia in five patients.

These two studies show that selinexor monotherapy has an antileukemic effect in AML; the toxicity is manageable, and hyponatremia is a common side effect.

### 10.2. Selinexor Can Be Combined with Various Forms of Conventional Intensive Chemotherapy; A Review of Important Nonrandomized Studies of Relapsed/Refractory and High-Risk AML

The design and hematological toxicity of these studies can be seen from Table 3 together with the number of patients, chemotherapy regimen, selinexor doses, and hematological toxicity. These clinical studies investigated the effect of combining selinexor with various strategies of antileukemic chemotherapy, and the large majority of them have concluded that the toxicity is acceptable (see Table 3). However, a recent randomized study concluded that combining selinexor with conventional cytotoxic treatment was associated with decreased long-term survival, and this was due to increased treatment-related mortality together with increased risk of AML relapse [109]. For these reasons, we have also included a summary of the hematological toxicity in Table 2 together with the antileukemic efficiency. We give a more detailed description of these important clinical studies of selinexor in AML therapy in the following text. The non-hematological toxicity in these studies was as would be expected (see Table 1), the most common severe toxicities being hyponatremia and severe neutropenic infections/sepsis and other common severe toxicities being anorexia, vomiting diarrhea, other electrolyte disturbances, and fatigue (see the text below). Finally, many of these nonrandomized studies included relatively few patients, and the studies mainly included patients with high risk/relapsed disease. However, we regard the relapse rate/survival in these studies to be as expected for such patients [3,4,5].

The MEC regimen. A small Phase I dose-escalating study including 23 relapsed/refractory adult patients below 60 years of age, investigated selinexor in combination with salvage chemotherapy with mitoxantrone, etoposide and cytarabine [108]. Most patients (78%) had received only one prior line of treatment, and patients with severe macular degeneration, uncontrolled glaucoma or markedly decreased visual acuity were excluded due to the risk of worsening of these conditions. Selinexor was distributed twice weekly for three consecutive weeks, and response/toxicity was evaluated after five weeks. The maximal tolerated selinexor dose was 30 mg/m^2^, and the dose-limiting toxicity was severe hyponatremia. Other severe non-hematological toxicities that occurred in at least 40% of patients were diarrhea/nausea/anorexia, edema, fatigue, hyperglycemia and hypoalbuminemia, and the most common grade ≥3 toxicities were febrile neutropenia, catheter-based infections, sepsis, and diarrhea. The overall response rate was 43% with six complete hematological remissions, two complete remissions with incomplete reconstitution and two additional patients without morphological signs of AML. The authors concluded that this combination was feasible for this group of AML patients.

Another report from this study investigated the expression of T cell checkpoint receptors and their ligands before and following selinexor combined with this induction treatment [117]. The frequency of pretreatment Gal9^+^ CD34^−^ cells at the time of AML diagnosis was significantly higher in patients with later treatment failure, and this finding correlated with increased TIM-3 expression on marrow-resident T cells after induction treatment. The Gal9/Tim-3 interaction is important for induction of T cell exhaustion, but it is not known whether this altered Gal9/Tim-3 expression (and possibly also increased T cell exhaustion) is seen only for selinexor-treated patients or is present also for treatment-failure patients receiving induction therapy without selinexor.

Cytarabine plus idarubicin induction. Selinexor in combination with idarubicin 10 mg/m^2^ for three days plus cytarabine 100 mg/m^2^ for seven days was investigated in 42 relapse/refractory AML patients [111]. Selinexor was administered twice weekly for four weeks; 27 patients received single doses of 40 mg/m^2^ and 15 patients 60 mg single doses. Prolonged aplasia (both neutropenia and thrombocytopenia grade 3/4), febrile neutropenia and severe diarrhea were frequent when using the higher dose, and the last 15 patients therefore received 60 mg flat single doses. In total, 20 patients achieved complete remission or complete remission with incomplete recovery (i.e., overall response rate 47.6%). The response rate for the reduced selinexor dose tended to be lower than with the higher dose, but the toxicity was also lower and the recommended dose for future studies was 60 mg flat twice weekly.

Cytarabine plus daunorubicin induction. This was a small single-arm Phase I clinical trial of selinexor combined with standard cytarabine 100 mg/m^2^/day plus daunorubicin 60 mg/m^2^/day 7 + 3 induction therapy in 21 newly diagnosed poor-risk patients (median age 69 years) [112]. Selinexor was given twice weekly for the first three consecutive weeks. The dose-finding Cohorts 1 (4 patients) and 2 (4 patients) received selinexor 60 and 80 mg, respectively, and an additional 13 patients thereafter received the selinexor 100 mg combination. Up to two consolidation cycles with selinexor similar to the induction treatment combined with cytarabine 5 days/daunorubicin 2 days were allowed for patients that achieved complete remission. The authors concluded that the maximal tolerated dose was not reached, and selinexor 80 mg was used for the 13 patients in the expansion part. The most common grade 3/4 nonhematological adverse events were febrile neutropenia (67%), diarrhea (29%), hyponatremia (29%), and sepsis (14%). The authors concluded that this combined treatment also had an acceptable toxicity.

Mitoxantrone plus high-dose cytarabine. A small Phase I dose escalation study including 20 patients with newly diagnosed or relapsed/refractory AML receiving selinexor (Days 2, 4, 9 and 11) combined with mitoxantrone and age-adjusted high-dose cytarabine [113]. No dose-limiting toxicities were observed, and the most common toxicities were diarrhea (40%), anorexia (30%), nausea/vomiting (25%), electrolyte abnormalities (30%), febrile neutropenia (70%), bacteremia (25%), cardiac toxicities (25%), and fatigue (25%). Serious events occurred in six patients, but the authors regarded the overall toxicities to be expected given the cytarabine/mitoxantrone regimen. In total, 10 patients achieved complete hematological remission. The recommended selinexor dose in combination with this intensive chemotherapy was 80 mg/day (~50 mg/m^2^/day) twice weekly.

FLAG-Ida. The safety and preliminary clinical activity of selinexor in combination with FLAG-Ida induction therapy was investigated in a very small Phase I study including 14 relapse/refractory AML patients (median age 52.5 years) [114]. Consolidation therapy was cytarabine 1 g/m^2^/day for six days combined with selinexor similar to the induction therapy. The examined selinexor doses were single weekly doses of 60 mg (3 patients), 80 mg (3 patients), and 100 mg (7 patients) on Days 5, 12 and 19. No dose-limiting toxicities were reported. Grade ≥3 non-hematologic adverse events occurred in 79% of patients, and 3 out of 14 patients had fatal adverse events. Five patients achieved complete remission, one of them with incomplete hematologic recovery. It is difficult to draw a firm conclusion about the safety of this selinexor combination based on such a small study, but high toxicity can be suspected.

Cladribine, cytarabine, and filgrastim induction. Selinexor combined with this treatment was investigated for 40 adults with relapsed/refractory AML (median age 55 years) [115]. The patients received selinexor 60 mg orally twice weekly for two weeks (i.e., Days 1, 5, 10 and 12). In total, 18 patients achieved complete remission or remission with incomplete recovery. Neutrophil and platelet recovery occurred after median times of 28 days (range 24–58 days) and 38 days (range 29–61 days), respectively. The most common non-hematological adverse event was gastrointestinal toxicity.

To conclude, the large majority of these studies included patients with relapsed/refractory AML receiving various forms of commonly used AML induction treatment. Many of the studies are small, and the observed response rates are relatively low, as would be expected for such patients. The hematological toxicity for such selinexor combinations was discussed in detail in Section 9, and the overall results described above suggest that the overall toxicity in these nonrandomized studies can be acceptable and justifies further randomized studies with a particular focus on hematological, infectious, and gastrointestinal toxicity.

### 10.3. Combination of Selinexor with AML-Stabilizing Decitabine Therapy

This Phase I dose escalation study assessed the safety and activity of oral selinexor in combination with the hypomethylating agent decitabine 20 mg/m^2^ in adults with relapsed/refractory and in elderly unfit (age ≥ 60 years) patients with untreated AML [118]. There were no dose limiting toxicities, and the recommended Phase 2 selinexor dose was 60 mg (~35 mg/m^2^) twice weekly. The most important Grade ≥3 toxicities included asymptomatic hyponatremia (68%), febrile neutropenia (44%), sepsis (44%), hypophosphatemia (36%), and pneumonia (28%). In 25 patients, the overall response rate was 40%. Modification of selinexor to a flat dose of 60 mg administered twice weekly for two weeks after decitabine improved tolerability and seemed to have an antileukemic effect in poor-risk AML.

### 10.4. Selinexor as Maintenance Treatment After Allogeneic Stem Cell Transplantation

This therapeutic strategy was investigated in a small study including 10 AML and 2 MDS patients [119]. The median time from transplantation to first selinexor dose was 97 days. Patients received selinexor 60 mg weekly, and they remained on selinexor for a median of 224 days. The only justified conclusion from this very small study is that selinexor monotherapy should possibly be used at lower weekly doses in post-transplant than in pre-transplant settings to avoid severe toxicity.

### 10.5. Selinexor Investigated in Randomized Studies; Decreased Survival for Elderly Patients Receiving Selinexor Combined with Conventional Intensive Induction Therapy

The results from two randomized studies are summarized in Table 4. One of these studies investigating conventional intensive chemotherapy observed a decreased remission rate as well as decreased survival for patients receiving selinexor, and the decreased survival was caused by increased relapse as well as fatal infections. For this reason, the table includes both hematological toxicity and antileukemic efficiency.

Newly diagnosed AML treated with cytarabine/anthracycline induction. The addition of selinexor therapy to intensive chemotherapy was investigated in an open label randomized Phase II study including 102 previously untreated AML patients above 65 years of age (median age 69 years) (Table 4) [109]. The patients received standard cytarabine 200 mg/m2/day plus daunorubicin 60 mg/m2/day 7 + 3 induction therapy with or without oral selinexor 60 mg twice weekly Days 1–24. In the second cycle, cytarabine 1000 mg/m2 twice daily for six days with or without selinexor was given. The rates of complete remission/complete remission without complete reconstitution were significantly higher in the control arm than in the investigational arm (80% versus 59%, *p* = 0.018), and after 18 months, both the event-free (45% versus 26%, *p* = 0.012) and overall survival (58% versus 33%, *p* = 0.009) rates were significantly higher for the control arm compared to the selinexor arm. The frequencies of Grade 3–4 adverse events were generally higher in the selinexor arm. The increased death rate could be explained by increased relapsed/refractory AML and infectious complications in the selinexor arm. The time until neutrophils >0.5 × 109/L was delayed in the selinexor arm after the induction cycle (29 versus 25 days, *p* = 0.0007) whereas platelet recovery did not differ, but neutrophil recovery did not differ for the second cycle. These observations strongly suggest that the dosing/toxicity of adding selinexor to conventional intensive AML-chemotherapy has to be carefully addressed, especially when treating elderly AML patients.

Relapsed/refractory AML; selinexor monotherapy versus investigators’ choice of AML-stabilizing treatment. A randomized Phase II study included patients above 60 years of age (n = 118, mean age 74 years) with relapsed/refractory AML evaluated selinexor 60 mg twice weekly monotherapy (n = 118) for patients receiving physician’s choice treatment (n = 57), i.e., the three possible therapeutic alternatives then being supportive care alone, low-dose cytarabine, or demethylating agents) (Table 4) [110]. The median overall survival did not differ significantly between selinexor versus physician’s choice (3.2 vs. 5.6 months). The selinexor patients had an increased incidence of adverse events; the most common Grade ≥3 events being thrombocytopenia, febrile neutropenia, anemia, and hyponatremia. However, it should be emphasized that the selinexor group included a higher number of high-risk patients with TP53 mutations, prior myelodysplastic syndrome and lower absolute neutrophil counts.

### 10.6. Combination of Selinexor with the Flt3 Inhibitor Sorafenib

A preliminary report on 14 patients included in an ongoing Phase IB clinical trial of selinexor combined with the Flt3 inhibitor sorafenib described complete/partial remissions in 6 of 14 patients with refractory AML who had received a median of 3 prior therapies (ClinicalTrials.gov: NCT02530476) [120].

## 11. Experimental Studies of XPO1 Inhibition Combined with Targeted AML Therapies

Several experimental studies have investigated the combination of Exportin 1 inhibitors with other targeted therapies (see Table 5 for an overview). These results suggest that the combination of selinexor with other targeted therapies should be further investigated in human AML.

### 11.1. Selexinor Combined with Bcl-2 Inhibition

A recent experimental study investigated the antileukemic effect of selinexor plus venetoclax on AML cell lines and primary AML cells [85]. The combination had synergistic proapoptotic effects, possibly due to inhibition of glycolysis and downregulation of DNA-replication-related genes; these in vitro effects were seen both in AML cell lines and primary AML cells. Another study showed that the two Exportin 1 inhibitors KPT-330/selinexor and KPT-8602/eltanexor decreases mRNA and protein levels of c-Myc, CHK1, WEE1, RAD51 and RRM2 [89]; both drugs also induced DNA damage that was enhanced by venetoclax possibly through inhibition of DNA damage repair.

### 11.2. Selinexor Combined with Demethylating Agents

Experimental studies have shown that selinexor combined with azacitidine has synergistic antiproliferative and proapoptotic effects in primary AML cells and AML cell lines [121]. This combined treatment down-regulated the expression of Exportin 1, eIF4E, and c-MYC, and additional knockdown of c-MYC further enhanced this effect. Moreover, the expression of Exportin 1 and eIF4E was often increased in primary AML cells, and patients with particularly high Exportin 1/elF4E expression had an adverse prognosis. Taken together, these observations suggest that the antileukemic effect of this drug combination is at least partly mediated by suppression of Exportin 1/eIF4E/c-MYC signaling.

Another study showed that sequential treatment of primary AML blasts with the hypomethylating agent decitabine priming followed by selinexor enhanced the antileukemic effects of selinexor [122]. This effect seemed to be mediated by re-expression of certain tumor suppressors (CDKN1A and FOXO3A) that are epigenetically silenced by DNA methylation and show cytoplasmic–nuclear trafficking regulated by Exportin 1. The combined treatment also increased the survival in an AML xenograft model compared with selinexor alone.

### 11.3. Selexinor Combined with Flt3 Inhibition by Sorafenib

A recent study investigated the combination of selinexor and the FLT3 inhibitor sorafenib [81] (see also Section 10.6). Selinexor induced apoptosis of AML cells harboring both ITDs and/or tyrosine kinase domain point mutations, and the selinexor/sorafenib combination showed synergistic proapoptotic effects. Five days of combined in vitro exposure to low doses (i.e., 5 to 10 nM) of each agent induced early myeloid differentiation of the AML cell lines MOLM13 and MOLM14 without cell death. Finally, the combined treatment also showed an antileukemic effect in a human FLT3-mutated xenograft model.

### 11.4. Selinexor Combined with PI3K-Akt-mTOR Inhibition

A study of non-Hodgkin’s lymphoma cells suggested that the combination of selinexor with everolimus shows increased anticancer effects [123]. Inhibition of PI3K-Akt-mTOR is also regarded as a possible therapeutic strategy in human AML, and combined treatment with Exportin 1 and PI3K-Akt-mTOR inhibition may be particularly effective in patients with high Exportin 1 levels because these patients also show high levels of several mediators of the PI3K-Akt-mTOR pathway [9].

### 11.5. Selinexor Combined with NF-κB/Proteasome Inhibitors

A previous study reported that cancer cells resistant to Exportin 1 inhibitors show increased expression of inflammation-related genes, including increased NF-κB transcriptional activity possibly mediated by reduced levels/effects of the cellular NF-κB inhibitor IκB-α [124]. Combined treatment with selinexor and proteasome inhibitors decreased NF-κB activity, sensitized resistant cells to Exportin 1 inhibition, and showed synergistic cytotoxicity in vitro and in vivo. Furthermore, selinexor inhibited NF-κB activity by blocking phosphorylation of the IκB-α and the NF-κB p65 subunits, thereby protecting IκB-α from proteasomal degradation and trapping IκB-α in the nucleus to suppress NF-κB activity. Therefore, combined Exportin 1 and NF-κB inhibition (e.g., through proteasome inhibition) should be further explored in human AML.

### 11.6. Targeting of Cellular Metabolism: Studies of Slinexor in Patients with DNMT3A Mutations

AML cells with DNA methyltransferase 3A (*DNMT3A*) mutation show increased expression of Exportin 1, and a recent study demonstrated that selinexor had stronger antiproliferative, proapoptotic, and cell-cycle-inhibitory effects in three DNMT3A mutated cell lines compared with their wild-type controls [53]. Furthermore, selinexor significantly inhibited the proliferation of subcutaneous tumors in *DNMT3AR882H* AML model mice, and primary cells with *DNMT3A* mutations were more sensitive to selinexor in chemotherapy-naive AML patients. Finally, RNA sequencing of selinexor-treated AML cells revealed that a majority of metabolic pathways were downregulated after selinexor treatment; the most significant change being in the glutathione metabolic pathway. Glutathione inhibitor L-buthionine-(S, R)-sulfoximine (BSO) significantly enhanced the apoptosis-inducing effect of selinexor in *DNMT3A* mutated AML cells.

### 11.7. Selinexor Combined with Topoisomerase II Inhibitors

Another study investigated the effect of selinexor combined with topoisomerase II inhibitors (idarubicin, daunorubicin, mitoxantrone, etoposide) on AML cells [90]. This combined treatment showed synergistic antileukemic effects in AML cell lines and primary patient samples as well as in the xenograft MV4-11 AML mouse model where the combination prolonged the survival of leukemic mice. Selinexor treatment resulted in nuclear retention of the topoisomerase II Topo IIα protein with increased sensitivity to idarubicin. Selinexor also caused a c-MYC-dependent reduction of DNA damage repair gene mRNA (Rad51, Chk1) and protein expression, which contributed to the increased sensitivity to topoisomerase II inhibitors.

These two studies illustrate that combination of Exportin 1 and metabolic inhibitors should be further considered at least for this/certain AML subsets.

## 12. Discussion

Exportin 1/XPO1 targeting is now tried in the treatment of several malignancies, including AML [101,102,103,104,105,106]. The available experience with selinexor monotherapy has shown that XPO1 inhibition has an anti-AML effect [106], but additional studies are needed to clarify the optimal use of this therapeutic strategy in AML.

Selinexor can have a wide range of adverse effects (Table 1 and Table 2, Figure 1); the toxicity is increased when selinexor is added to standard intensive AML therapy [109], including the hematological toxicity. This seems to be especially true for induction treatment, whereas the risk seems lower for consolidation cycles [109,114]; there may also be a difference depending on the type of chemotherapy (see Table 2), and it has been suggested that the risk is increased especially for anthracycline-based regimen [115]. Finally, the risk of severe selinexor-associated neutropenia/infections seems to be dose-dependent [110,111,112]. Additional clinical studies must clarify whether/how the risk of severe selinexor toxicity varies between combinations. The question of hematological toxicity is of particular importance, whereas the risk of several other (often dose-dependent) toxicities can often be handled through optimal supportive care [108,115].

Many previous studies have included high-risk relapsed/refractory AML patients. Further studies are needed to clarify whether the anti-AML effect of selinexor/Exportin 1 inhibition is seen, especially for certain subsets of patients (e.g., elderly patients), and thus whether this strategy should be avoided or employed with reduced dosage in these patients. Alternatively, this treatment should be used only in certain parts of the AML treatment (e.g., consolidation/maintenance/posttransplant) for patients with susceptibility to severe toxicity. Additional studies are also needed to clarify whether Exportin 1 inhibition should be combined with certain forms of targeted therapies.

The question of hematological toxicity is particularly important in combination therapy because even selinexor monotherapy has a risk of anemia, neutropenia and thrombocytopenia. The only available randomized study of elderly patients receiving anthracycline/cytarabine-based induction therapy suggests that prolonged neutropenia with increased mortality can be observed when selinexor is added [119]. The question of thrombocytopenia is also of particular importance because selinexor inhibits thrombopoietin signaling and thereby may be toxic to early megakaryopoiesis [125].

Venetoclax is now used in routine AML treatment in combination with demethylating agents for elderly and unfit patients [126]. In vitro studies of AML cell lines and primary AML cells suggest that the venetoclax plus selinexor combination had synergistic antileukemic effects [85]. However, this combination has until now only been investigated in a small study of four myeloma patients, and no unexpected toxicity was then observed [127]. The combination should therefore be considered in AML, but future clinical studies must carefully address the question of toxicity.

A recent study demonstrated that prolonged exposure is necessary for an optimal anti-AML effect of selinexor [87]. Repeated doses over 14–21 days has been commonly used in previous clinical studies (see Table 3 and Table 4), but it is not known whether the interval between single doses is too long to achieve an optimal effect. The antileukemic efficiency may therefore be strengthened by certain new Exportin inhibitors that seem to have a longer half-life together with a better toxicity profile [87].

Previous experimental studies have described multiple effects of Exportin 1 and Exportin 1 inhibition in human AML cells (see Table 1), and this is not surprising when taking into account the large number of Exportin 1 cargo molecules. This diversity of functional and molecular effects suggests that the dominant antileukemic mechanism of Exportin 1 inhibition also differs between patients and depends on the AML cell genotype/phenotype. For this reason, the optimal use/combinations of Exportin 1 inhibitors may also differ between AML patient subsets. Furthermore, it is not known which of the molecular effects are directly caused by the altered cytoplasmic levels of Exportin 1 cargo proteins and which effects should possibly be regarded as secondary or indirect because they reflect either (i) effects induced by altered levels or compartmentalization of the cargo molecules or (ii) functions/mechanisms due to molecular/cellular responses to the altered levels/distribution/function of cargo molecules.

Homoharringtonine is used in the treatment of AML; it is used both as part of intensive therapy [128,129,130] but also in combination with demethylating agents plus BCL2 targeting [131]. The exact mechanism for its anti-AML activity is not known in detail, but a recent study described that homoharringtonine directly targets Exportin 1 by binding to the molecular cleft involved in binding of cargo molecules [132]. This drug can thereby inhibit the Exportin 1 nuclear export function. The possible use of homoharringtonine as an alternative Exportin 1 inhibitor can therefore be further investigated.

## 13. Conclusions

Exportin 1 inhibition has an anti-AML effect, but for the first-generation inhibitor selinexor the toxicity seems to be a problem, especially hematological toxicity and central nervous system toxicities like anorexia and weight loss. Experimental studies including xenograft models suggest that the second-generation inhibitor KPT-8602 has a greater antileukemic efficiency and an improved tolerability profile [89,133]. However, it should be emphasized that Exportin 1 inhibition should not be regarded as a part of routine AML therapy and should be tried only for AML patients included in clinical trials. One should also remember that AML is a heterogeneous disease (this is true even for AML cell expression of Exportin 1) and that the antileukemic effect of Exportin 1 inhibition will probably depend on the AML cell phenotype and vary between patient subsets.

## Figures and Tables

**Table 1:** Molecular interactions of Exportin 1 in AML cells, a summary of important observations in experimental studies. The table presents the cellular functions or molecular mechanisms (left column) and the effects on these functions/mechanisms together with the references (right column).

Function or Molecule	Effect on Cellular Functions and/or Molecular Mechanisms
Proliferation Apoptosis	Relatively strong antiproliferative and proapoptotic effects in *DNMT3* mutated AML cells; this has been observed in cell lines, xenograft models, and primary AML cells [53]. Exportin 1 inhibition has antileukemic effects in *NPM1* mutated AML cells [78]. Exportin 1 inhibition has proapoptotic effects in *TET2* mutated AML cells without evidence of DNA damage [79]. Exportin inhibition has strong antileukemic effects in *MLL-AF9*-driven murine AML [80]. *FLT3-ITD* AML shows high Exportin 1 levels [9], and exportin inhibition has antileukemic effects in human *FLT3*-mutant AML [81]. AML cells with *p53* mutations show decreased susceptibility to Exportin 1 inhibition [9]. Exportin 1 inhibition has a cytotoxic effect on the minority of AML-initiating cells within the hierarchically organized AML cell population [82]. Apoptin is a cytoplasmic protein that functions as a regulator of apoptosis, cell cycle progression and DNA damage responses; its expression is associated with adverse prognosis in AML and Exportin 1 inhibition causes nuclear retention of this protein [83].
Cell cycle	Exportin 1 inhibition can induce cell cycle arrest [84].
Differentiation	Exportin 1 inhibition can induce myeloid AML cell differentiation [84].
Intracellular signaling	Exportin 1 inhibition activates PI3Kγ-AKT signaling in AML cells by upregulation of the P2RY2 purinergic receptor; inhibition of this downstream signaling potentiates the antileukemic effects of Exportin 1 inhibition in murine and human AML [80]. Several proteins that correlate with Exporin 1 expression are components of the AKT signaling patway; they include AKT, its upstream PI3Kp85 and phospho-PTEN, and downstream phospho-BAD (Ser112, Ser136) as well as 14-3-3 [9].
Metabolism	Exportin 1 inhibition causes downregulation of several metabolic pathways, especially glutathione metabolism, and combined treatment with Exportin 1 and glutathione inhibition can have synergistic antileukemic effects [53]. Exportin inhibition enhances antileukemic effects of BCL2 inhibition by inhibition of glycolysis [85].
Transcription Epigenetic regulation	Exportin inhibition enhances the antileukemic effects of BCL2-inhibition via downregulation of genes involved in DNA replication [85]. Exportin 1 can bind to chromatin and thereby accumulate at *HOX* cluster regions, recruit nucleoporin-fusion proteins and finally activate *HOX* genes [86]. Furthermore, Exportin 1 inhibition causes irreversible downregulation of *HOX* genes in *NPM1* mutated AML [87]. Exportin 1 can bind to chromatin and then recruit the CALM-AF10 fusion protein; this leads to transcriptional/epigenetic activation of *HOXA* genes that are important for maintenance and progression of the leukemia [88].
DNA damage	Exportin 1 inhibition can induce DNA damage probably through inhibition of DNA damage repair [89,90].
NPM1 mutation	Exportin 1 levels of the AML cells are only marginally higher in patients with NPM1 mutations compared to other AML patients [9]. Both classic and exon 5 mutations of *NPM1* encode proteins that bind to Exportin 1, resulting in an aberrant cytoplasmic dislocation of the abnormal NPM1 protein that is not observed for normal NPM1 [91,92,93]. This aberrant NPM1 dislocation causes high expression of *HOX* genes [87]. Mutant *NPM1* seems to maintain the leukemic state through this *HOX* gene activation; relocation of NPM1 from the cytoplasm to the nucleus by Exportin 1 inhibition in *NPM1* mutated AML then leads to *HOX* downregulation and AML cell differentiation [94].
FLT3, FLT3-ITD	Exportin 1 inhibition can activate FLT3 and its downstream mediators MAPK or AKT; combined Exportin 1 and FLT3 inhibition has synergistic pro-apoptotic effects and also causes AML cell differentiation, possibly due to nuclear retention of ERK, AKT, NFκB, and FOXO3a [81]. The antileukemic effect has also been demonstrated in a human *FLT3*-mutated xenograft model [81]. Another study described downregulation of the FLT3 protein as a result of Exportin 1 inhibition [84].
BCL2 family	Exportin 1 and BCL2 inhibition have synergistic proapoptotic effects [95]. This synergism is partly mediated by MCL1; Exportin 1 inhibition then decreases mRNA and protein levels of c-Myc, CHK1, WEE1, RAD51, and RRM2 [89]. Exportin 1 inhibition decreases MCL-1 protein levels; the inhibition can also prevent MCL-1 binding to BIM but further enhance the increased BCL2 binding to BIM in AML cells [95].
TP53	p53 levels are particularly high for AML cells with high Exportin 1 and low MDM2 levels [9]. Exportin 1 inhibition increases cellular p53 protein levels and activates the p53 target genes *TP53I3*, *GDF15*, *MDM2*, *PUMA*, *ZMAT3,* and *p21* [9]. Exportin 1 inhibition seems to especially induce the FLp53 isoform, i.e., it alters the p53 isoform profile in human AML cells and seems to synergize with MDM2 inhibition to induce p53 expression and thereby apoptosis in AML ells [9]. The proapoptotic effect of Exportin 1 inhibition in human AML seems to depend on p53, whereas the antiproliferative effects depend on other mechanisms [9].
Topoisomerase II	Aberrant nuclear export and cytoplasmic localization of TOPO IIα (topoisomerase II) can lead to chemoresistance in a subset of AML; Exportin 1 inhibition will then result in nuclear retention of the Topo IIα protein and thereby increased sensitivity to TOPO II inhibitors. Exportin 1 inhibition then result in c-MYC-dependent reduction of DNA repair gene expression (*RAD51* and *CHK1*) that probably contributes to the increased sensitivity to TOPO II inhibitors [90].

**Table 2:** Side effects registered for at least 10% of AML patients receiving selinexor in combination with conventional chemotherapy [108,109,110,111,112,113,114,115,116,117]. The table lists adverse events reported in at least 10% of the patients in at least one of the referred studies. The table is based on those available studies with a detailed description of the toxicity.

**Constitutional**
Fatigue, weight loss, anorexia, malaise/weakness
**Gastrointestinal**
Diarrhea, nausea, constipation, oral mucositis, vomiting, gastroesophageal reflux, dysgeusia, abdominal pain, perianal discomfort
**Neurological/Psychiatric**
Dizziness, depression, insomnia, muscle weakness, asthenia, headache, muscle/bone pain
**Vascular/Cardiac/Pulmonary**
Sinus tachycardia, QT prolongation, heart failure, hypotension, hypertension, edema, catheter-associated thrombosis, syncope, dyspnea, neuropathy, pleural effusion, cough
**Electrolytes and Nutrition**
Anorexia, dehydration, hyponatremia, hyperglycemia, hypoalbuminemia, hypokalemia, hypophosphatemia, hypocalcemia, hypomagnesemia
**Renal and hepatic toxicity**
Increased creatinine, increased bilirubin, increased liver enzymes
**Skin**
Alopecia, rash, dry skin
**Infectious**
Febrile neutropenia, sepsis, lung infection, catheter-related infection
**Hematological**
Thrombocytopenia grade ≥3, neutropenia grade ≥3, lymphopenia, coagulopathy

**Table 3:** Hematological toxicity in clinical AML studies where selinexor has been combined with intensive chemotherapy. The table presents the observations from important nonrandomized studies with a summary of patient characteristics and chemotherapy (left), selinexor treatment (middle left), and the hematological toxicity/recovery together with the antileukemic efficiency (middle right) and the references (right) [111,112,113,114,115].

Patients and Treatment	Selinexor	Neutrophil/Platelet Recovery Antileukemic Efficiency	Ref
Refractory/relapsed adult AML patients (n = 42). Idarubicin 10 mg/m^2^ Days 1, 3 and 5; cytarabine 100 mg/m^2^ Days 1–7.	Selinexor either 40 mg/m^2^ or 60 mg flat twice weekly for 4 weeks.	**Recovery:** Grade ≥3 adverse events were 26/42 for thrombocytopenia and 18/42 for neutropenia. Recommended single dose for future Phase II studies 60 mg flat. **Efficiency:** 20/42 complete remission or remission with incomplete recovery.	[111]
High-risk AML patients (n = 21, median age 69 years). Daunorubicin 60 mg/m^2^ Days 1–3; cytarabine 100 mg/m^2^ Days 1–7.	Most patients received selinexor 60 mg (4 patients 80 mg) single doses on Days 1, 3, 8, 10, 15 and 17.	**Toxicity:** For 10 patients reaching complete remission median time to neutrophils >50 × 10^9^/L was 26 days (range 18–45) and median time to platelets >50 × 10^9^/L 35 days (range 25–77). **Efficiency:** 8/19 patients with complete remission or remission with incomplete recovery.	[112]
Newly diagnosed and relapsed/refractory AML (n = 20, median age 61 years). Cytarabine 2/3 g/m^2^ and mitoxantrone 20/30 mg/m^2^ on Days 1 and 5 (lower dose when age >70 years).	Single dose 80 mg (17/20) or 80 mg Days 2, 4, 9 and 11.	**Toxicity:** Median time to complete remission 37.5 days (range 26–50 days); median time until neutrophils > 0.5 × 10^9^/L 31 days (range 22–48 days), time to platelets >20 × 10^9^/L 25 days (range 19–38 days). **Efficiency:** 10/20 complete remissions.	[113]
Relapsed/refractory AML (n = 14, median age 53 years). Induction: Fludarabine 30 mg/m^2^ Days 1–4, idarubicin 10 mg/m^2^ Days 1–3, cytarabine 2 g/m^2^ Days 1–4, G-CSF 300 μg/m^2^ Days 1–5. Consolidation: cytarabine 1 g/m^2^ Days 1–6.	Selinexor one dose weekly for three weeks, Escalating doses of 60/80/100 mg (7/12 largest dose)	**Toxicity:** Two early deaths, 12 patients evaluated for safety. Median time to neutrophils >0.5 × 10^9^ /L was 40 days (range 22–63 days) during induction and 15 days (13–57 days) during consolidation, median time to platelets >20 × 10^9^ /L for induction 21 days (range 0–41 days) and consolidation 18 days (6–50 days). **Efficiency:** 4/14 complete remissions, 1 of them with incomplete recovery.	[114]
Refractory or first relapse AML (n = 40, median age 56 years). Cladribine 5 mg/m^2^ Days 4–8, cytarabine 2000 mg/m^2^ Days 4–8, G-CSF 300 μg Days 3–8.	Selinexor 60 mg Days 1, 5, 10 and 12.	**Toxicity:** Seven patients without morphological signs of AML failed to recover platelets before consolidation/allotransplantation. Prolonged neutropenia was not observed. **Efficiency:** 18/40 patients with complete remission or remission with incomplete recovery.	[115]

**Table 4:** Hematological toxicity in clinical AML studies where selinexor has been combined with intensive chemotherapy. The table presents the observations from the available randomized studies with a summary of patient characteristics and chemotherapy (left), selinexor treatment (middle left) and the hematological toxicity/recovery together with the antileukemic efficiency (middle right) and the references (right) [109,110].

Patients and Treatment	Selinexor	Neutrophil/Platelet Recovery Antileukemic Efficiency	Ref
Previously untreated patients (n = 102, median age 69 years) Induction: Daunorubicin 60 mg/m^2^ daily Days 1–3, cytarabine 200 mg/m^2^ Days 1–7	With or without 60 mg twice weekly Days 1–24	**Toxicity:** Selinexor associated with increased infectious Grade 3–4 toxicity (57% versus 37%) and prolonged time to neutrophil recovery >0.5 × 10^9^/L (median 29 versus 25 days, *p* = 0.007). Platelet recovery did not differ.	[109]
Consolidation: Cytarabine 1000 mg/m^2^ twice daily Days 1–6.	With or without 60 mg twice weekly Days 1–24	No difference in hematological recovery. **Efficiency:** The selinexor arm showed decreased complete remission/remission without complete reconstitution, event-free and overall survival.	
Relapsed/refractory AML (median age 74 years). Selinexor (n = 118) versus supportive care alone or combined with either low-dose cytarabine or demethylating agent (n = 57).	60 mg twice weekly for 21-day, 28-day cycles	**Toxicity:** Neutropenia and thrombocytopenia Grade ≥3 were frequent in both the selinexor and physician’s choice groups. Five out of seven patients with fatal events possibly/probably related to selinexor had fatal infections. **Efficiency:** The complete remission/complete remission without complete reconstitution rates were significantly higher in the control arm (80% versus 59%, *p* = 0.018); after 18 months, both event-free (45% versus 26%, *p* = 0.012) and overall survival (58% versus 33%, *p* = 0.009) were higher for the controls.	[110]

**Table 5:** Combination of Exportin 1 inhibitors with targeted therapies in AML. The table gives a summary of available studies and presents the targeted cellular mechanism/molecular target, the paharmacological agent used and the observed effects. For references and additional information, please see the text.

Cellular Mechanism/Target	Agent	Effect
Regulation of apoptosis, altered metabolic regulation	Venetoclax (anti-BCL2)	Synergistic proapoptotic effects, inhibition of glycolysis [85,89]
DNA hypomethylation	Azacitidine, decitabine	Synergistic antiproliferative and proapoptotic effects [121,122]
Intracellular signaling	Sorafenib, Flt3 inhibition	Synergistic proapoptotic effects, myeloid differentiation [81]
	Everolimus, PI3K-Akt-mTor inhibition	Increased antileukemic effects [123]
Proteasome inhibition	Carfilzomib	Decreased NF-κB activity, synergistic cytotoxicity [124]
Glutathione metabolism	L-buthionine-(S, R)-sulfoximine	Enhanced proapoptotic effects [53]
Topoismerase II targeting	Idarubicin, daunorubicin, mitoxantrone, etoposide	Synergistic antileukemic effects, decreased DNA repair [90]

## Data Availability

Not applicable.
